# Exploring the Cellular and Molecular Landscape of Idiopathic Pulmonary Fibrosis: Integrative Multi-Omics and Single-Cell Analysis

**DOI:** 10.3390/biomedicines13092135

**Published:** 2025-09-01

**Authors:** Huanyu Jiang, Shujie Wang, Fanghui Zhong, Tao Shen

**Affiliations:** School of Basic Medical Sciences, Chengdu University of Traditional Chinese Medicine, Chengdu 611137, China; jhybuula@stu.cdutcm.edu.cn (H.J.); wangshujie@stu.cdutcm.edu.cn (S.W.); zhongfanghui@stu.cdutcm.edu.cn (F.Z.)

**Keywords:** idiopathic pulmonary fibrosis, single-cell sequencing, machine learning, diagnostic biomarkers, mendelian randomization

## Abstract

**Background/Objectives**: Idiopathic pulmonary fibrosis (IPF) is a progressive disease characterized by lung scarring, impaired function, and high mortality. Effective therapies to reverse fibrosis are lacking. This study aims to uncover the molecular mechanisms of IPF, explore diagnostic biomarkers, and identify therapeutic targets. **Methods**: Multi-omics data were integrated to identify biomarkers with causal associations to IPF using Mendelian randomization and transcriptomic analysis. Machine learning was employed to construct a diagnostic model, and single-cell transcriptomic analysis determined gene expression patterns in fibrotic lung tissue. **Results**: Seven core genes (*GREM1*, *UGT1A6*, *CDH2*, *TDO2*, *HS3ST1*, *ADGRF5*, and *MPO*) were identified, showing strong diagnostic potential (AUC = 0.987, 95% CI: 0.972–0.987). These genes exhibited distinct distribution patterns in fibroblasts, endothelial cells, epithelial cells, macrophages, and dendritic cells. **Conclusions**: This study highlights key genes driving IPF, involved in pathways related to metabolism, immunity, and inflammation. However, their utility as fluid-based biomarkers remains unproven and requires protein-level validation in prospective cohorts. By integrating genomic, immunological, and cellular insights, it provides a framework for targeted therapies and advances mechanism-based precision medicine for IPF.

## 1. Introduction

As a unique subtype of interstitial lung diseases (ILDs), idiopathic pulmonary fibrosis (IPF) has the greatest mortality rate and the shortest survival duration. Its etiology is unknown, and its global incidence is roughly 58.7 cases per 100,000 people. Half of the patients diagnosed with IPF succumb to the disease within three to five years [1,2]. Patients often experience debilitating dyspnea, which severely limits mobility, and in the late stages, their quality of life is reported to be lower than that of most malignancies [3]. To date, the exact etiology and molecular mechanisms underlying IPF remain poorly understood. A widely accepted concept of IPF involves dysregulated wound healing following alveolar epithelial cell injury. During this process, fibroblast-to-myofibroblast differentiation occurs, leading to excessive fibroblast proliferation and scar tissue accumulation. This results in the overaccumulation of extracellular matrix (ECM) within the lung parenchyma, ultimately causing pathological remodeling of lung structure [4,5,6].

Recent years have witnessed substantial advancements in understanding the pathophysiological mechanisms of IPF, elucidating its genetic predisposition and distinctive gene transcription profiles [7,8]. However, existing studies have predominantly focused on single molecules or pathways, lacking a systematic integration of interactions across multi-omics levels [9]. Traditional approaches often adopt a single-molecule strategy [10,11]. The significant heterogeneity of IPF complicates the ability of single-level statistics to comprehensively represent the disease’s complexity. Although genome-wide association studies (GWASs) have revealed genetic risk loci related with the beginning of IPF, there is inadequate evidence connecting these loci to functional alterations in proteins [12]. Similarly, although proteomic studies have identified differentially expressed proteins in the lung tissue of IPF patients, the dynamic interactions between these proteins, genetic background, and immune states remain poorly understood.

Plasma proteins are regarded as optimal biomarkers since they may be collected non-invasively and effectively represent normal and pathological conditions. Nonetheless, prior research has been confined to basic protein–phenotype correlations, overlooking the complex connections among plasma proteins, genetic variants, and transcriptional regulatory networks that affect IPF. Fundamental inquiries persist without resolution: Are these proteins directly implicated in essential pathogenic processes, like ECM production and fibroblast activation, or are they simply secondary byproducts? How can causal relationships between IPF and differentially expressed proteins be distinguished through multi-omics integration?

In this study, we incorporate multi-omics data focused on circulating plasma proteins to systematically characterize the molecular networks of IPF. We aim to identify proteins causally linked to IPF and uncover actionable therapeutic targets. Candidate targets were validated for their expression and distribution in lung tissue from IPF patients using RNA sequencing, laying the foundation for the development of blood-based biomarkers. Finally, single-cell transcriptomics was employed to investigate cell type-specific regulatory mechanisms and to analyze the expression and distribution of candidate targets among various cell populations.

## 2. Materials and Methods

### 2.1. Data Source

The IPF GWAS dataset utilized in this study was derived from a meta-analysis of 7 independent case-control studies, comprising 5159 cases of IPF and 27,459 controls [13]. All participants were unrelated individuals of European ancestry. The diagnosis of IPF cases was based on the protocols delineated by the American Thoracic Society (ATS) and the European Respiratory Society (ERS). Detailed documentation of data quality control and sample selection methods was provided in the original studies. The research adhered strictly to ethical standards, with written informed consent obtained from all participants and approval granted by relevant institutional review boards, in accordance with the principles outlined in the Declaration of Helsinki.

Genetic summary statistics related to plasma proteins in individuals of European ancestry were obtained from the study by Ferkingstad et al. [14]. To ensure data quality and relevance, the pQTL data were filtered using several stringent criteria. Only associations that reached genome-wide significance (*p* < 5 × 10^−8^) and were independent (r^2^ < 0.001) were included. Additionally, only cis-pQTLs were considered, and associations with an F-statistic greater than 10 were retained. Applying these criteria resulted in the identification of 241,653 SNPs associated with 4288 proteins.

We retrieved transcriptomic datasets containing lung tissue samples from IPF patients and healthy controls from the GEO database (https://www.ncbi.nlm.nih.gov/geo/; accessed on 28 June 2025). GSE150910 includes transcriptomic profiles of lung samples from 103 IPF patients and 103 controls, with participant demographics showing a mean age of 59.9 years and 48.6% males. GSE213001 comprises lung tissue samples from 41 IPF cases and 62 controls, with a mean age of 58.7 years and 69.7% males. Additionally, we obtained single-cell RNA sequencing data of lung tissue from IPF patients and controls, also from the GEO database. The GSE136831 dataset includes samples from 32 IPF patients and 28 controls, providing high-resolution insights into cell type-specific expression patterns.

### 2.2. Mendelian Randomization (MR) and Functional Enrichment Analysis

LD estimation was based on the European population dataset from the 1000 Genomes Project, and SNP filtering was performed using PLINK v2.0.0 alpha. To ensure the robustness of the results, various MR approaches were employed. The primary findings were derived using the inverse variance-weighted (IVW) approach. For cases with only a single instrumental variable (IV), the Wald ratio method was applied. Horizontal pleiotropy was assessed using the MR-Egger regression approach, and the MR-PRESSO method was utilized to correct for any bias caused by genetic pleiotropy. Heterogeneity among genetic variants was evaluated using the Cochrane Q test. Finally, the MR-Steiger test was conducted to verify the correct directionality of each exposure factor, ensuring consistency with the expected causal direction. Two-sample MR (TSMR) analyses were performed using the R package “TwoSampleMR” (version 0.6.6) in R 4.3.2. Gene ontology (GO) and Kyoto Encyclopedia of Genes and Genomes (KEGG) enrichment analyses were performed using the R package “clusterProfiler” (version 4.10.1) to investigate the biological functions and signaling pathways related to proteins identified as causally connected with IPF by MR analysis.

### 2.3. Workflow for Identification of Differentially Expressed Genes (DEGs)

Differential expression analysis was conducted on the GSE150910 discovery dataset and the GSE213001 validation dataset utilizing the Limma R package (version 3.1.8). *p*-values were adjusted for multiple comparisons using the false discovery rate (FDR) method, and genes with adjusted *p*-values (P.adj) < 0.05 and |log2 FC| > 1 were identified as DEGs. To identify genes with consistent expression levels and causal relationships, the upregulated genes were intersected with those identified by MR analysis with an OR > 1. Similarly, downregulated genes were intersected with genes identified by MR analysis with an OR < 1. These intersections yielded genes whose expression levels aligned with their inferred causal relationships. For exploratory unsupervised clustering, expression values of the intersected genes were Z-scored per gene, and hierarchical clustering was performed using Spearman correlation distance (1 − ρ) and Ward.D2 linkage. The number of clusters (K) was selected by maximizing average silhouette width over K = 2–4. Per-gene differences between clusters were evaluated on Z-scored values using two-sided Wilcoxon rank sum tests, followed by Benjamini–Hochberg FDR correction. Principal component analysis (PCA) was applied to the same Z-scored matrix of intersected genes to obtain a low-dimensional visualization of sample structure. PCA was computed from the covariance matrix (genes centered and scaled). PCA was used for visualization only; no clustering or hypothesis testing was conducted in the PCA space.

### 2.4. Identification and Validation of Diagnostic Gene Signatures

The diagnostic performance of key genes was assessed using receiver operating characteristic (ROC) curves and the area under the curve (AUC) in both the discovery dataset (GSE150910) and the validation dataset (GSE213001). Key gene selection was further refined using least absolute shrinkage and selection operator (LASSO) regression, implemented in the “glmnet” R package (version 4.1-8). Ten-fold cross-validation was performed based on the optimal log (Lambda) value to identify candidate genes. Subsequently, the “e1071” R package (version 1.7-16) was employed to develop a support vector machine recursive feature elimination (SVM-RFE) model, which evaluated the significance of candidate genes and identified the ideal gene combination based on error rate and accuracy. The conclusive gene set was established by intersecting the outcomes of the LASSO regression and SVM-RFE models.

### 2.5. Development of a Predictive Nomogram and Gene Interaction Network Analysis

A nomogram was constructed using the “rms” R package (version 6.8-0) to assess the predictive performance of machine learning-identified biomarkers for IPF. A calibration curve was created utilizing the “regplot” R package (version 1.1) to confirm the precision of the predictive model. The clinical utility of the model was assessed through decision curve analysis (DCA) using the “ggDCA” R package (version 1.1). Additionally, the GeneMANIA platform (https://genemania.org/; accessed on 28 June 2025) was employed to construct a gene interaction network, enabling the exploration of interactions among key genes. This network provided insights into the functional relationships and potential pathways involving the identified biomarkers.

### 2.6. Single-Cell RNA Sequencing (ScRNA-Seq) Data Processing

Prior to analysis, quality control was performed on the ScRNA-seq data. Cells with mitochondrial gene expression > 25% or gene counts outside the range of 300–10,000 were excluded. The top 2000 highly variable genes for each sample were normalized. Sequencing features were aligned to the reference genome (GRCh38). Dimensionality reduction was performed using the RunPCA function, where the number of principal components was set to 20. Cell clustering was then conducted using the FindNeighbors and FindClusters functions with a resolution of 0.6. To integrate all samples and mitigate batch effects, we applied RunHarmony. Batch mixing was assessed on the resulting embedding using the local inverse Simpson’s index (iLISI) and the fraction of cross-batch neighbors in a k-NN graph (k = 50). Nonlinear dimensionality reduction was performed using the uniform manifold approximation and projection (UMAP) method in the Seurat package (version 4.4.0). This approach mapped high-dimensional cell data into a two-dimensional space, grouping cells with similar expression patterns while separating cells with distinct expression profiles. Cell types were annotated based on commonly reported lung tissue markers from previous studies. Furthermore, intercellular interactions among annotated cell types were explored using the CellChatDB human database, which provides ligand–receptor interaction data.

## 3. Results

### 3.1. MR Analysis of the Relationship Between Plasma Circulating Proteins and IPF

**Figure 1:**
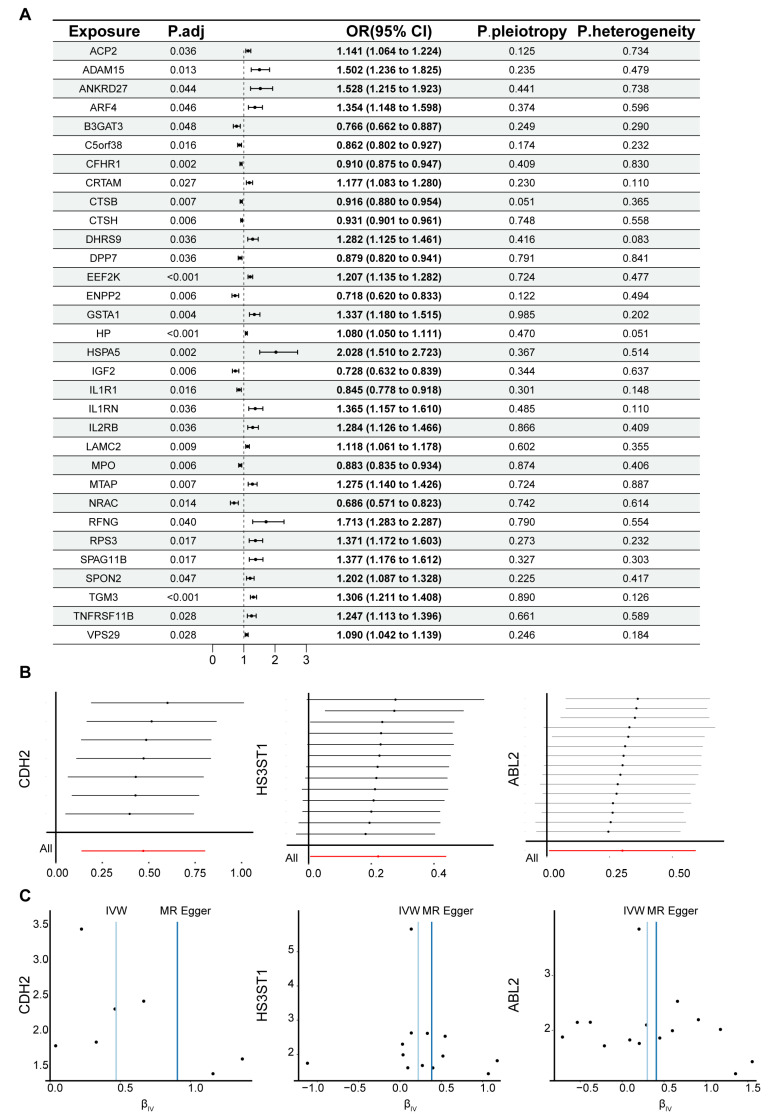
Circulating proteins identified by MR with causal associations with IPF. (**A**) The 32 proteins with significant causal associations after FDR correction. (**B**) Results of the leave-one-out sensitivity analysis for the association between proteins and IPF (only partial results are shown). Each horizontal gray line represents the IVW causal estimate (point) and its 95% CI (line) obtained after excluding one SNP instrument at a time. (**C**) Comparison of effect estimates across different MR methods (only partial results are shown).

A total of 429 proteins were identified to have significant causal relationships with IPF, of which 32 remained significant after FDR correction. Among these, 11 proteins were identified as protective factors for IPF, while 21 proteins were identified as risk factors (Figure 1A). To assess the robustness of the results, we employed the Q-test to examine heterogeneity and used Egger’s intercept to evaluate horizontal pleiotropy. To further validate the findings from the MR analysis, we also conducted leave-one-out analysis (Figure 1B) and a Steiger filter test. These analyses confirmed that all proteins identified by MR were robust candidates with significant causal relationships with IPF. Additionally, the randomness test validated that the MR analysis adhered to the second principle of MR analysis (Figure 1C).

### 3.2. Functional Enrichment Analysis for MR-Identified Plasma Circulating Proteins

**Figure 2:**
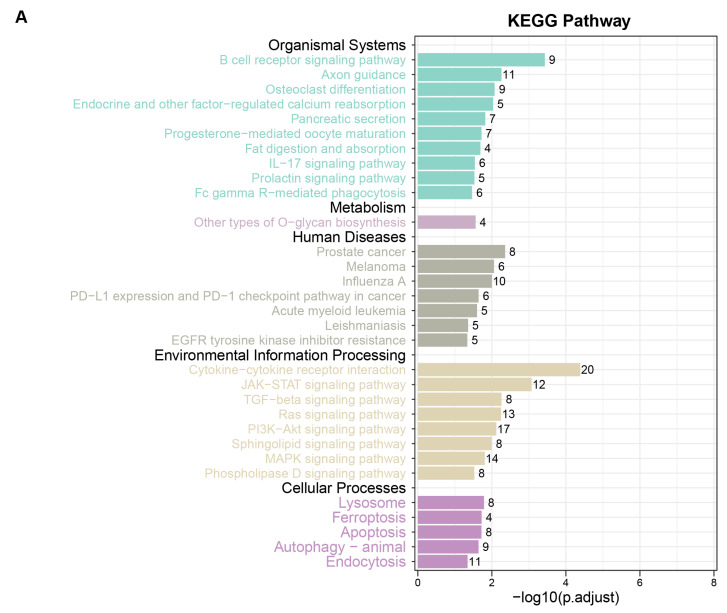

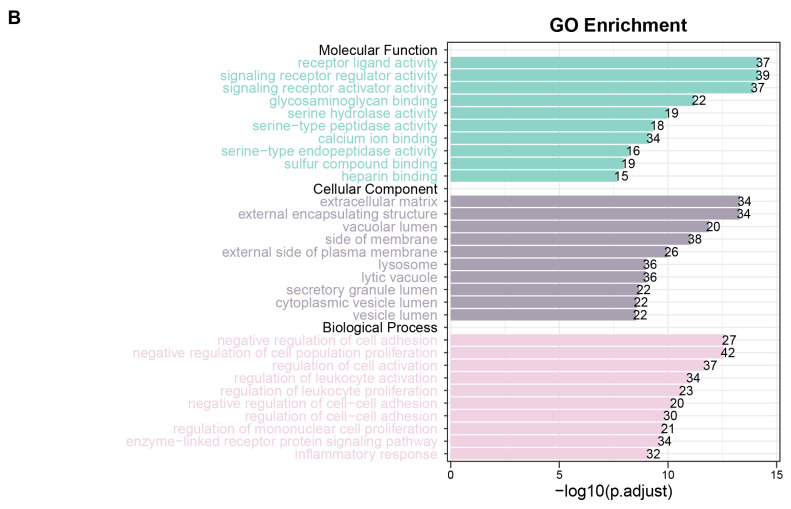
Functional enrichment analysis. (**A**) KEGG pathway enrichment analysis showing 31 significantly enriched pathways. (**B**) GO enrichment analysis, displaying the top 10 most significantly enriched terms for biological process (BP), molecular function (MF), and cellular component (CC).

We performed functional enrichment analysis on the proteins identified by MR with nominal associations to IPF to explore their potential biological functions and signaling pathways. KEGG pathway enrichment analysis identified 31 significantly enriched pathways, which could be categorized into 5 major groups, namely organismal systems, metabolism, human diseases, environmental information processing, and cellular processes. The results suggest that these proteins may be involved in metabolic changes, cell death, and inflammatory responses during the development and progression of IPF. Notably, pathways, such as ferroptosis and the TGF-beta signaling pathway, which have been widely reported in IPF, were also identified (Figure 2A).

GO molecular function (GO-MF) enrichment analysis revealed that these genes contributed to receptor–ligand activity, highlighting their importance in signal transduction and metabolic regulation. For cellular components, the most significantly enriched term was the ECM, suggesting that these genes are likely involved in ECM formation and degradation, which play crucial roles in IPF. In terms of biological processes, these genes were mainly associated with cell–cell interactions, immune defense mechanisms, and other related processes (Figure 2B).

### 3.3. Identification of Differentially Expressed Genes (DEGs)

**Figure 3:**
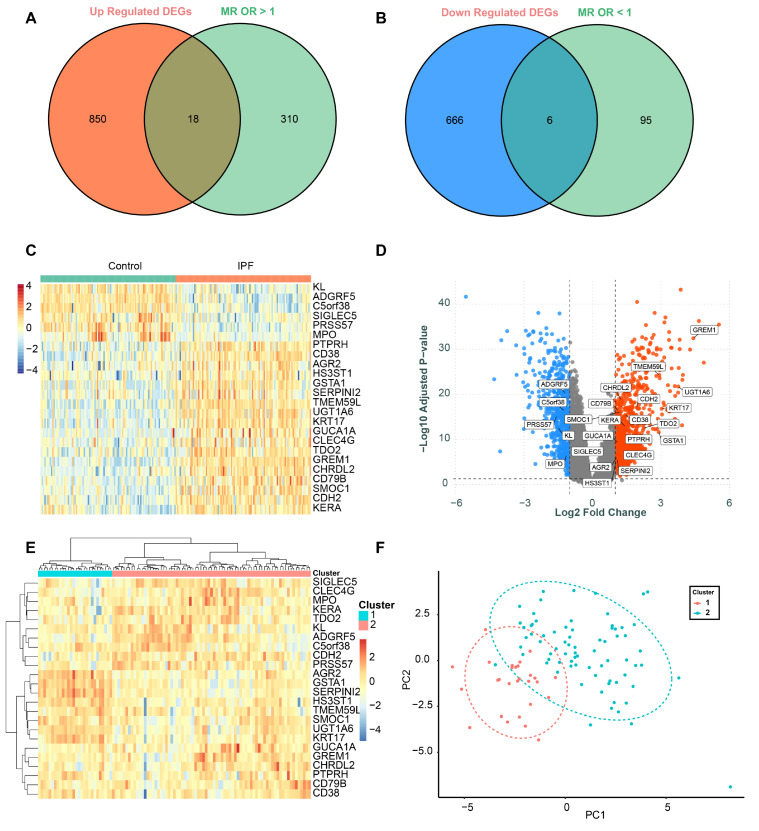
Overlapping genes identified through differential expression analysis and MR results. (**A**) The intersection of upregulated DEGs and MR-identified genes with OR > 1. (**B**) Intersection of downregulated DEGs and MR-identified genes with OR < 1. (**C**) The expression levels of the 24 overlapping genes (18 upregulated and 6 downregulated) across control and IPF samples. (**D**) The logFC- and −log10-adjusted *p*-values of all differentially expressed genes, with the 24 overlapping genes highlighted. Red dots, significantly upregulated genes (log2 FC > 0 and FDR-adjusted *p* < 0.05); blue dots, significantly downregulated genes (log2 FC < 0 and FDR-adjusted *p* < 0.05); gray dots, not significant. (**E**) Unsupervised clustering of IPF cases using the 24-gene matrix (Cluster 1: n = 28; Cluster 2: n = 75). (**F**) PCA of the 24-gene matrix.

Differential expression analysis identified 868 upregulated genes and 672 downregulated genes. By intersecting the upregulated genes with MR-identified genes having OR > 1, we obtained 18 overlapping genes. Similarly, intersecting the downregulated genes with MR-identified genes having OR < 1 yielded six overlapping genes (Figure 3A,B). Figure 3C shows the expression levels of these 24 genes across all samples in GSE150910, while Figure 3D illustrates the logFC- and −log10-adjusted *p*-values of these genes.

We performed exploratory unsupervised clustering of IPF cases using the 24 intersected genes. The optimal partition was K = 2 by average silhouette width (K = 2: 0.24; K = 3: 0.235; K = 4: 0.224), yielding 2 groups (Cluster 1: n = 28; Cluster 2: n = 75) (Figure 3E,F). Per-gene comparisons between clusters on Z-scored values identified coordinated differences after Benjamini–Hochberg correction, with higher *SERPINI2*, *GSTA1*, *AGR2*, *HS3ST1*, *UGT1A6*, and *KRT17* in Cluster 1 (*SERPINI2* meanZ: 1.19 vs. −0.44, FDR = 5.94 × 10^−12^; *GSTA1*: 0.99 vs. −0.37, FDR = 4.52 × 10^−11^; *AGR2*: 1.03 vs. −0.39, FDR = 7.59 × 10^−10^), and higher *ADGRF5*, *KERA*, *CLEC4G*, and *SIGLEC5* in Cluster 2 (*ADGRF5* meanZ: −0.72 vs. 0.27, FDR = 2.42 × 10^−5^; *KERA*: −0.64 vs. 0.24, FDR = 1.65 × 10^−4^; *CLEC4G*: FDR = 3.81 × 10^−4^; *SIGLEC5*: FDR = 4.75 × 10^−4^). A PCA of the 24-gene matrix illustrates the separation between the 2 groups (Figure 3F). Given the modest silhouette, this stratification should be considered exploratory and warrants validation in independent cohorts.

**Figure 4:**
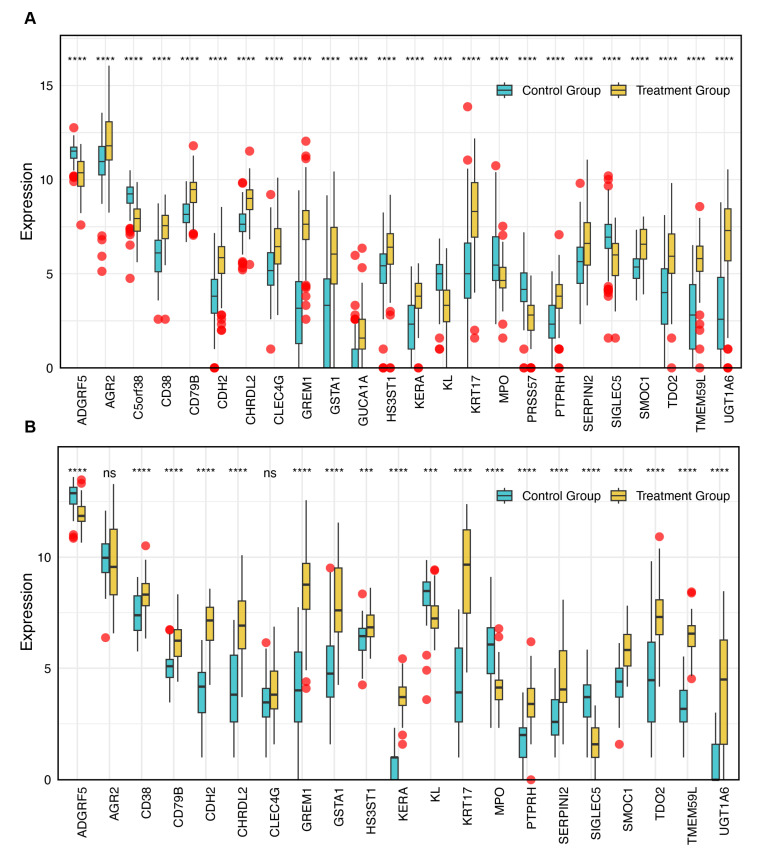
Expression levels of 24 overlapping genes in the discovery (GSE150910) and validation (GSE213001) datasets. Red dots denote individual observations (including outliers) for both groups (**A**) The expression levels of the 24 overlapping genes in lung tissues from IPF patients and healthy controls in the discovery dataset. (**B**) The expression levels of the same 24 genes in the independent validation dataset. Statistical significance is indicated as follows: ns = not significant, *** *p* < 0.001, and **** *p* < 0.0001.

Subsequently, the expression levels of these 24 genes in lung tissues from IPF patients and healthy controls were visualized using bar plots (Figure 4A). Validation was performed in the GSE213001 independent dataset, where *C5orf38*, *PRSS57*, and *GUCA1A* were not available due to dataset limitations. The results showed that the expression levels of *AGR2* and *CLEC4G* were not significantly different in the validation set, which was inconsistent with the findings in the discovery set. However, the expression levels and significance of all other genes were successfully validated, showing consistency with the results from the discovery set (Figure 4B).

### 3.4. Evaluation of Diagnostic Performance for DEGs

**Figure 5:**
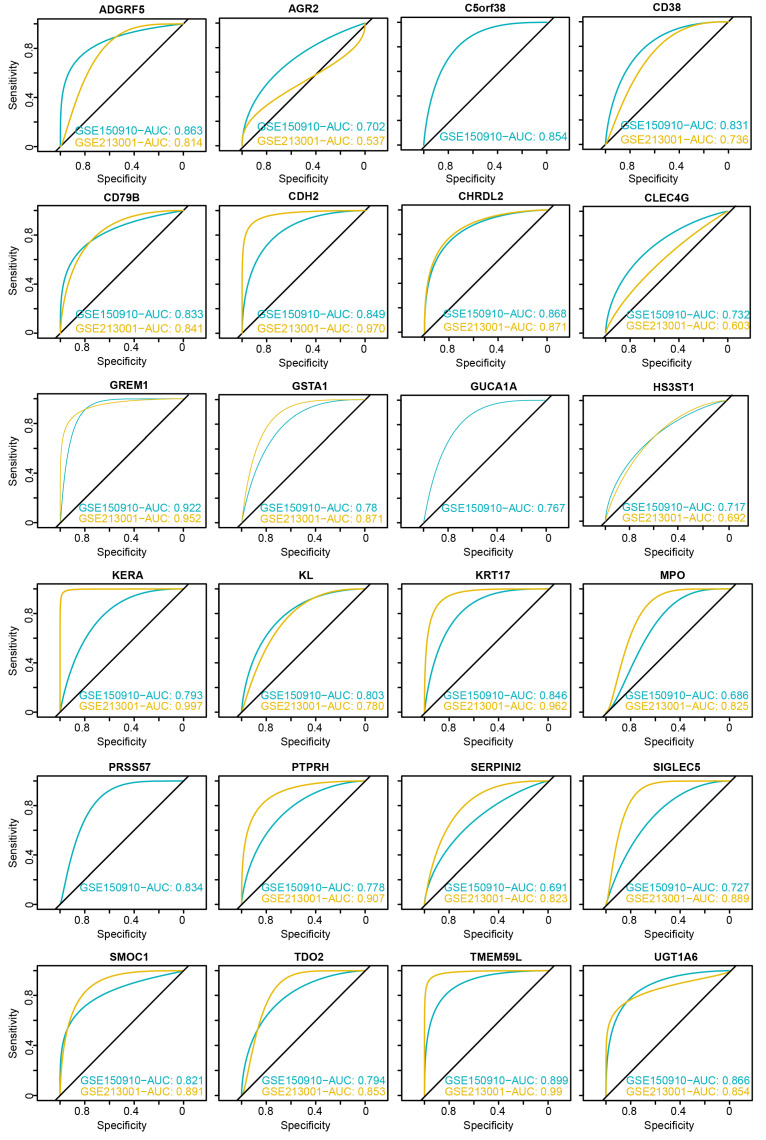
ROC curve analysis of candidate genes in the discovery and validation datasets. C5orf38, PRSS57, and GUCA1A were not included in the validation dataset.

After identifying the candidate genes, we evaluated their diagnostic performance by calculating the AUC values in both the discovery and validation datasets (Figure 5). In the GSE150910 discovery dataset, all candidate genes achieved an AUC greater than 0.68. In the GSE213001 validation dataset, the AUC values for most genes were consistent with those in the discovery dataset. Notably, GREM1 exhibited an AUC greater than 0.9 in both datasets, while *ADGRF5*, *CD79B*, *CDH2*, *CHRDL2*, *KRT17*, *SMOC1*, *TMEM59L*, and *UGT1A6* showed AUC values exceeding 0.8.

### 3.5. Identification of Core Genes and Construction of a Robust Diagnostic Model

**Figure 6:**
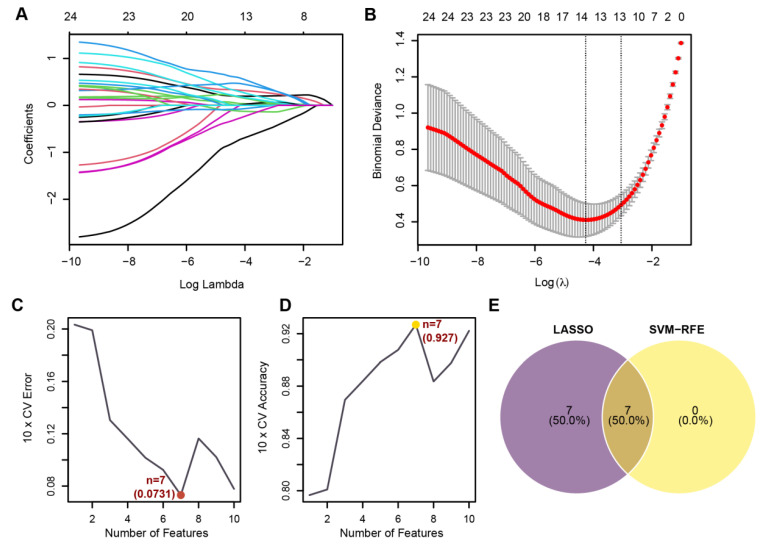

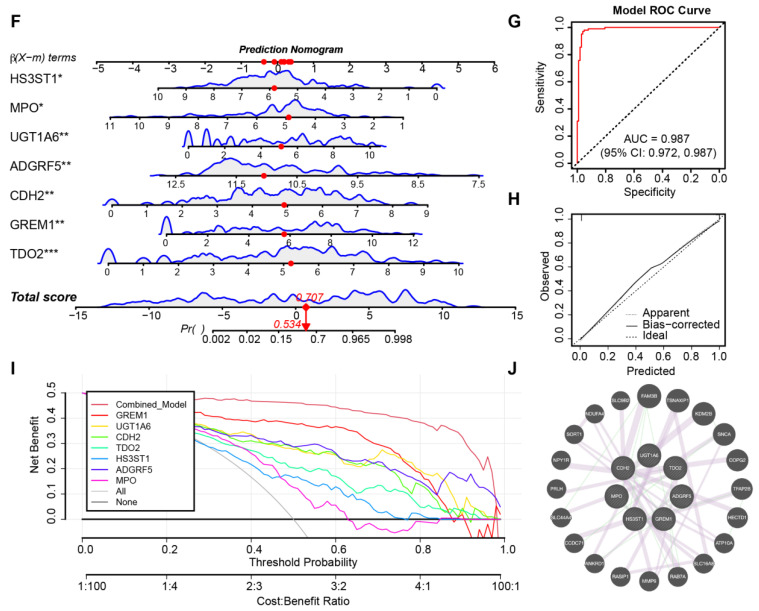
Identification of core genes and evaluation of the diagnostic model. (**A**) Coefficient profiles of Lasso regression, showing the shrinkage of feature coefficients with increasing log lambda values; each colored line represents a distinct predictor and its coefficient along the regularization path. (**B**) Selection of the optimal Lambda value using 10-fold cross-validation, minimizing binomial deviance and identifying 7 features; red points/curve show the mean cross-validated binomial deviance at each log(λ), and gray vertical bars denote ±1 SE around the mean. (**C**) The 10-fold cross-validation error of the SVM-RFE method decreases with the number of selected features, with the lowest error achieved at 7 features. (**D**) The 10-fold cross-validation accuracy of SVM-RFE peaks when 7 features are selected. (**E**) The overlap of features selected by Lasso regression and SVM-RFE, resulting in 7 shared features. (**F**) Nomogram constructed using the 7 core genes, representing their contributions to IPF prediction. * *p* < 0.05, ** *p* < 0.01, *** *p* < 0.001 (**G**) ROC curve analysis of the diagnostic model, showing excellent performance, with an AUC of 0.987 (95% CI: 0.972–0.987). (**H**) Calibration curve of the diagnostic model, indicating strong agreement between predicted and observed probabilities. (**I**) DCA comparing the net benefit of the combined model to individual genes across a range of threshold probabilities. (**J**) Network generated with GeneMANIA using default parameters; edge types include co-expression and inferred genetic interactions. Relationships are predictive in nature and not necessarily direct physical interactions in IPF.

To further refine the candidate genes, Lasso regression (Figure 6A,B) and SVM-RFE (Figure 6C,D) were applied to the discovery dataset. By intersecting the results of both methods (Figure 6E), seven core genes were identified as disease-related feature genes, namely *GREM1*, *UGT1A6*, *CDH2*, *TDO2*, *HS3ST1*, *ADGRF5*, and *MPO*. These genes hold potential as biomarkers for disease diagnosis and provide a foundation for further research. Using these seven core genes, a diagnostic model was constructed based on a nomogram (Figure 6F). The model yielded outstanding diagnostic performance, with an AUC of 0.987 (95% CI: 0.972–0.987) in the ROC curve analysis (Figure 6G). Calibration curves demonstrated strong consistency between predicted and observed probabilities, confirming the model’s reliability (Figure 6H). Additionally, decision curve analysis (DCA) revealed that the combined model offered a higher net benefit compared to individual genes across a range of threshold probabilities (Figure 6I). Finally, putative relationships among the seven core genes were explored using the GeneMANIA network. GeneMANIA integrates co-expression and interaction evidence from curated scientific literature and large-scale datasets. The resulting network in Figure 6J reflects predicted/co-supported associations (e.g., co-expression, inferred genetic interactions) rather than IPF-specific, experimentally validated physical interactions. These hypotheses provide context for potential pathway involvement but will require targeted experimental validation in future studies.

### 3.6. ScRNA-Seq Analysis Reveals Distinct Expression Patterns of Core Genes in Normal and Fibrotic Lung Tissues

**Figure 7:**
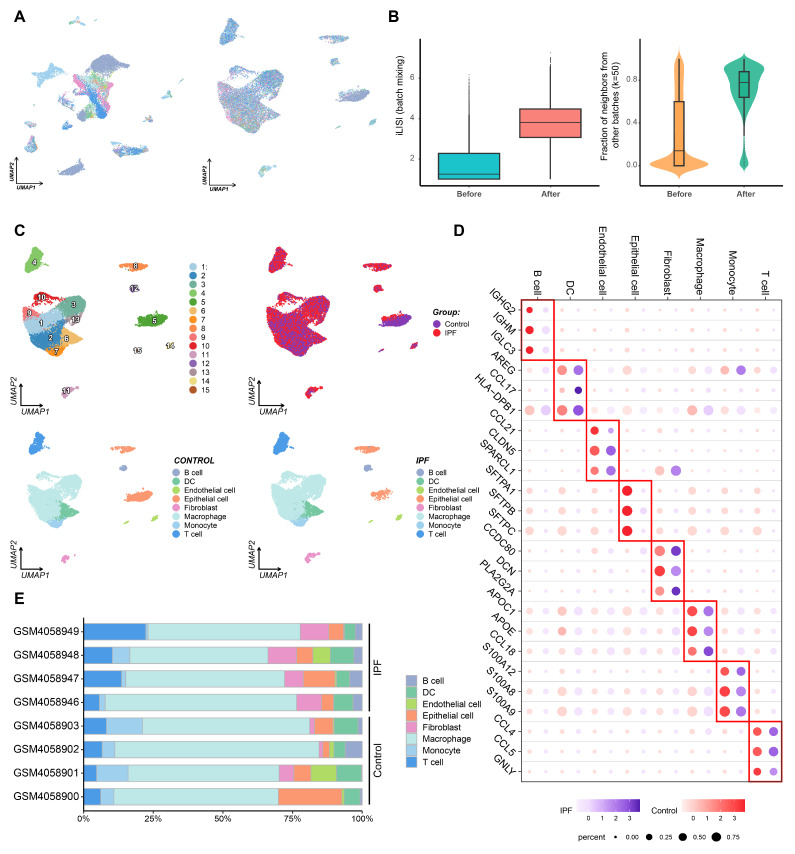

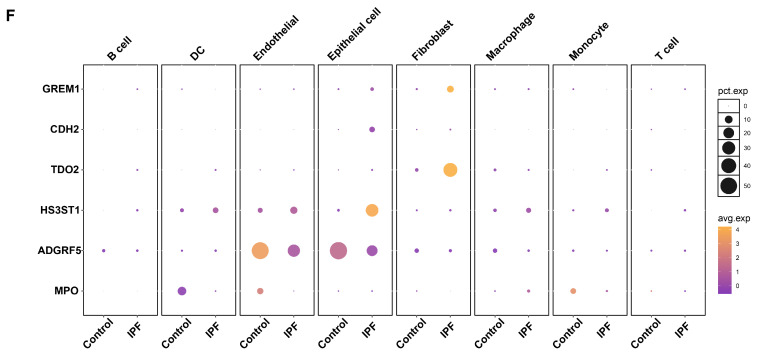
Cellular composition and expression patterns of core genes in normal and fibrotic lung tissues. (**A**) UMAPs colored by sample before (**left**) and after (**right**) Harmony integration showing improved cross-batch mixing post-integration; each color represents one sample. (**B**) **Left**: iLISI (batch mixing) before vs. after Harmony. **Right**: fraction of neighbors from other batches (k = 50) before vs. after Harmony. (**C**) UMAP analysis of scRNA-seq data identifies eight major cell clusters. (**D**) The expression of known marker genes for the eight identified cell types in both normal and IPF samples. (**E**) The proportion of each cell type in individual samples from both control and IPF groups. While the dataset includes 32 IPF patients and 28 controls, the chart displays representative data from 4 control and 4 IPF samples for clarity. (**F**) The expression levels and proportions of the seven core genes across different cell types in control and IPF samples.

To investigate the specific expression patterns of the seven core genes in normal and fibrotic lung tissues, we analyzed scRNA-seq data from patients with IPF. After data integration with Harmony, we first validated that batch effects were effectively mitigated. UMAPs colored by sample showed markedly improved cross-batch mixing after integration compared with the pre-integration embedding (Figure 7A). Quantitatively, batch mixing increased as evidenced by higher iLISI values after Harmony versus before, and by a larger fraction of cross-batch neighbors in k-NN graphs (k = 50) post-integration (Figure 7B). UMAP analysis identified eight major cell clusters, including B cells, dendritic cells (DCs), endothelial cells, epithelial cells, fibroblasts, macrophages, monocytes, and T cells (Figure 7C). Significant differences in cellular composition were observed between normal lung tissue and fibrotic lung tissue. The expression of known markers for these eight major cell types in the normal and IPF groups is shown in Figure 7D. We further examined the proportion of each cell type in different sample groups (Figure 7E). Macrophages were the most abundant cell type across all samples. As expected, the proportion of fibroblasts was significantly increased, while the proportion of epithelial cells was markedly decreased in IPF samples compared to normal controls.

We next analyzed the expression levels and distribution patterns of the seven core genes across different cell types. Notably, these genes exhibited distinct distribution characteristics between healthy controls and IPF lung tissues, particularly in epithelial cells, endothelial cells, and fibroblasts. Specifically, *GREM1* and *TDO2* showed significantly increased expression levels and proportions in fibroblasts from IPF patients, with *TDO2* being the most prominent. *CDH2* was predominantly upregulated in epithelial cells in IPF patients. *HS3ST1* was expressed in DCs, endothelial cells, and epithelial cells in both groups, but its expression was significantly higher in epithelial cells from IPF patients compared to healthy controls. ADGRF5 was highly expressed in endothelial and epithelial cells in healthy controls. In contrast, *MPO* was mainly expressed in DCs, endothelial cells, and monocytes in healthy controls, but its expression was significantly downregulated in IPF patients (Figure 7F).

### 3.7. Cell–Cell Interaction Patterns Highlight Macrophage- and Fibroblast-Associated Communication in IPF

**Figure 8:**
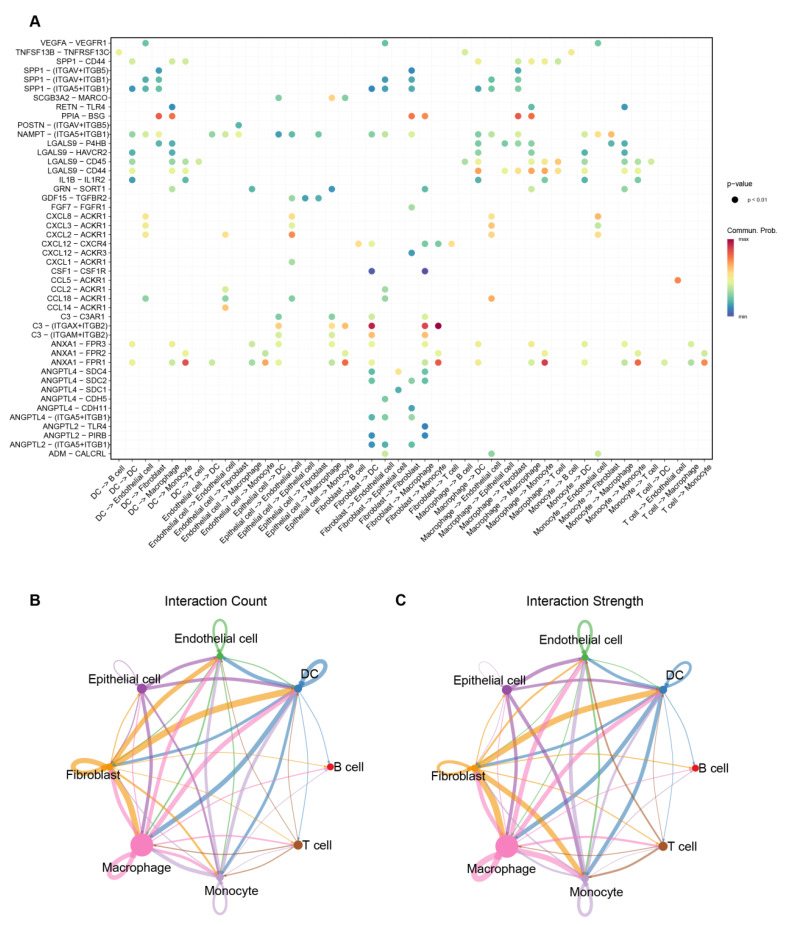
Cell–cell communication analysis indicates macrophage- and fibroblast-associated interaction patterns in IPF. (**A**) Significant ligand–receptor interactions among cell types. (**B**) The interaction count between different cell types; each node (cell type) has a unique color, and edges inherit the color of the source/ligand-expressing cell (or the dominant contributor) (**C**) The interaction strength between cell types; each node (cell type) has a unique color, and edges inherit the color of the source/ligand-expressing cell (or the dominant contributor).

To further investigate the intercellular interactions within the single-cell dataset, we performed cell–cell communication analysis (Figure 8A). The inferred network placed macrophages among the most connected cell types by degree and interaction strength. Macrophages demonstrated dense communication with epithelial cells, endothelial cells, and fibroblasts. As key effector cells in the progression of IPF, fibroblasts were closely connected with endothelial cells, DCs, and monocytes. Notably, fibroblasts communicated with monocytes and DCs predominantly through the C3/ITGAX + ITGB2 signaling pathway, forming an extensive interaction network.

## 4. Discussion

This multi-omics study, combining MR, transcriptomics, machine learning, and ScRNA-seq data analysis, systematically elucidates the molecular mechanisms underlying IPF. We identified seven key genes that are significantly differentially expressed in lung tissue and causally associated with IPF at the plasma protein level. Furthermore, we characterized the potential biological functions and regulatory pathways of these genes and analyzed their expression patterns and cellular distributions at the single-cell level. This study not only deepens our understanding of the pathogenesis of pulmonary fibrosis but also establishes a predictive model for IPF clinical diagnosis and identifies potential therapeutic targets, providing a valuable foundation for future clinical interventions.

Consistent with prior single-cell and review studies, we observed transcriptomics-inferred, highly connected cell–cell communication patterns between macrophages and fibroblasts in fibrotic IPF tissue, along with prominent macrophage-associated compositional features [15,16]. Macrophages exhibit substantial functional plasticity, shifting along a continuum from classically activated (M1) to alternatively activated (M2) states in response to microenvironmental cues, accompanied by corresponding molecular markers and pathway changes [17,18].

In general, M1-associated programs are linked to proinflammatory responses and early injury reactions (iNOS, IL-1β, IL-12, and TNF-α), whereas M2-associated programs are linked to anti-inflammatory/repair processes and fibrosis-related matrix remodeling (Arg1, Fizz1/RELM-α, and Mrc1/CD206) [19]. M2-related features are more prominent in IPF lungs and are associated with excessive ECM deposition and disease severity [20,21,22]. Mechanistically, M2 programs are commonly driven by type 2 immune pathways, notably IL-4/IL-13 signaling that activates the JAK1/STAT6 and PI3K/AKT axes [23]. In addition, M2-like macrophages can secrete CCL18 to stimulate fibroblast collagen production and, through collagen binding, form a positive feedback loop, indicating active macrophage–fibroblast crosstalk in scar regions [24].

However, these observations are associative and do not establish that macrophages are initiating or singular causal drivers in IPF. Discriminating contributory/amplifying roles from reactive consequences will require directional evidence, such as temporally and spatially resolved longitudinal sampling, spatial multi-omics validation, selective perturbations targeting specific macrophage subsets or key pathways (e.g., the CCR2 axis, IL-4/IL-13–STAT6, ADORA2B, and SPP1/CCL18 circuits), and functional co-culture/perturbation assays with fibroblasts [16].

Among the seven key genes identified, *GREM1*, *UGT1A6*, *CDH2*, *TDO2*, and *HS3ST1* were found to be risk factors for IPF, whereas *ADGRF5* and *MPO* were identified as protective factors. Single-cell data analysis revealed that *GREM1* and *TDO2* are highly expressed in fibroblasts of IPF patients. Studies have shown that fibroblasts in fibrotic lung tissue exhibit characteristics of high expression of secretory proteins, such as CCDC80, CTHRC1, COL6A1, FBLN2, FSTL1, and GSN [25]. Notably, some of these proteins (e.g., FSTL1) have been demonstrated to exacerbate lung fibrosis by promoting EMT [26,27].

The GREM1 protein, a member of the TGF-β superfamily, has been shown to promote the migration and proliferation of normal lung cells while inducing EMT and EndMT [28,29,30]. Moreover, *GREM1* is closely associated with the fibrotic progression of multiple tissues, including the lungs, liver, eyes, and skin [31,32]. A recent study in a silica-induced mouse silicosis model has identified a class of inflammatory proliferative fibroblasts characterized by high *GREM1* expression. These fibroblasts mediate the TGF-β1/GREM1/PPP2R3A signaling pathway through their downstream target gene *PPP2R3A*, thereby promoting early fibrotic changes. These findings further support the potential of *GREM1* as a predictive biomarker for pulmonary fibrosis [33]. *GREM1* is also found at markedly higher levels in the serum of IPF patients compared to patients with other ILDs and healthy controls [34].

scRNA-seq enables us to identify interactions between cell surface receptors and their ligands. Cell–cell communication analysis revealed a high intensity and quantity of interactions between fibroblasts and macrophages. *GREM1* has been shown to promote macrophage polarization toward an M2-like phenotype [35]. Therefore, we hypothesize that the high levels of M2-like macrophages may be closely related to the secretion of *GREM1* by IPF-associated fibroblasts. This represents a novel cellular mechanism potentially driving pulmonary fibrosis and remodeling.

Therapeutic strategies targeting *GREM1* have shown potential in the treatment of pulmonary fibrosis. For instance, targeting *GREM1* or its regulatory pathways (USP11 inhibition) has demonstrated reduced fibrosis and improved outcomes in animal models. Certain drugs, such as demethyleneberberine, have exhibited promising efficacy and safety profiles [36].

TDO2 is a key enzyme in tryptophan metabolism, responsible for converting tryptophan into kynurenine. The kynurenine/tryptophan ratio is significantly elevated in the blood of patients with fibrotic lung diseases [37]. Consistent with our findings, previous research has demonstrated that *TDO2* is highly expressed in alveolar fibroblasts of IPF patients, while it is almost undetectable in normal lung fibroblasts [38]. Kynurenine can activate the aryl hydrocarbon receptor (AHR) on the surface of DCs, particularly in CD103+ DCs. This activation leads to increased production of pro-inflammatory cytokines, such as IL-6 and IL-17, which further promote fibrosis. Additionally, TDO2, as a key enzyme in the metabolism–immune axis driving pulmonary fibrosis, plays a crucial bridging role in the communication between fibroblasts and DCs. Based on this mechanism, targeting the *TDO2* pathway may offer a novel strategy for the treatment or monitoring of pulmonary fibrosis.

CDH2 (N-cadherin) is another key molecular driver of tissue remodeling in IPF. Its elevated expression is closely associated with fibroblast activation and the production of fibrosis markers, such as type I collagen and α-SMA. Studies have shown that reducing *CDH2* expression in fibroblasts significantly decreases the expression of these fibrosis markers, highlighting the essential role of *CDH2* in the complete fibrotic response [39,40]. The transcription factor *FOXF1* inhibits fibrosis by suppressing the cadherin switch from *CDH2* to *CDH11* in myofibroblasts. Loss of *FOXF1* leads to increased *CDH11* expression, triggering more aggressive fibrotic behavior. However, restoring *CDH2* expression or blocking *CDH11* function can effectively alleviate fibrosis [41]. In our study, *CDH2* was found to be highly expressed in epithelial cells, suggesting that it may play an important role in EMT. The high expression of *CDH2* may promote the functional remodeling of epithelial cells into mesenchymal cells, further facilitating fibroblast activation and the fibrotic response.

Previous studies have shown that MPO and its antibodies (MPO-ANCA) play important roles in the development of microscopic polyangiitis (MPA) and ILD. ILD occurs in 52% of ANCA-associated vasculitis patients as a preceding condition and in 39% as a concurrent condition [42]. Although the association between MPO-ANCA and ILD has been widely studied, its specific relationship with IPF remains less explored. A few studies report that the MPO-ANCA positivity rate in IPF and ILD patients is 4% and 15%, respectively, suggesting that MPO-ANCA positivity may represent a distinct subtype of IPF [43]. Additionally, the usual interstitial pneumonia (UIP) pattern is more common in MPO-ANCA-positive ILD cases, suggesting potential mechanistic overlap with IPF. It is noteworthy that these patients’ prognoses are similar to or slightly better than those with IPF [44]. Our study further found that *MPO* is highly expressed in DCs and endothelial cells in healthy lung tissue, which may be closely associated with its roles in oxidative stress regulation, immune homeostasis maintenance, and endothelial integrity protection. However, in IPF patients, DCs almost completely lose *MPO* expression, suggesting significant functional alterations, such as a shift from an immunoregulatory to a profibrotic phenotype. Similarly, the reduced expression of *MPO* in endothelial cells may impair their antioxidant capacity, promoting EndMT and accelerating fibrosis progression.

ADGRF5 (GPR116) is an adhesion G protein-coupled receptor that plays a critical role in lung function regulation and immune homeostasis. Studies have shown that the loss of *ADGRF5* leads to chronic airway inflammation, characterized by increased mucus secretion, subepithelial fibrosis, and elevated markers of type 2 immune responses. Additionally, fibrosis-related genes, such as *Tgfb1* and *Col1a1*, are significantly upregulated in *ADGRF5* knockout models [45]. The activation of *ADGRF5* depends on the tethered agonist mechanism, a process essential for maintaining pulmonary surfactant homeostasis. Disruption of this activation can lead to pathological changes resembling fibrosis [46]. Although there is insufficient evidence directly linking *ADGRF5* to human pulmonary fibrosis, its roles in airway inflammation, fibrosis-related gene expression, and immune regulation suggest that *ADGRF5* may serve as a potential therapeutic target or biomarker for fibrotic lung diseases [47].

We identified seven core genes with robust diagnostic performance in lung tissue. These signatures may be clinically actionable. Composite gene scores constructed from these markers could assist diagnostic enrichment and risk stratification when combined with clinical variables (e.g., FVC decline, DLCO, AE-IPF). Given that several genes encode secreted or cell-surface-associated products or lie in druggable pathways (e.g., GREM1, TDO2, and CCR2/IL-4–IL-13–STAT6-related axes), targeted panels could support patient selection and pharmacodynamic monitoring in early-phase trials. Furthermore, selected markers may be adapted to minimally invasive matrices (peripheral blood or BALF) to enable screening or disease activity monitoring, contingent on analytical validation and stability testing.

Several limitations should be noted in this study. First, IPF is a highly heterogeneous and progressive disease. Factors, such as the location of sample collection and the degree of fibrosis in different datasets, may influence the study results. Distinct driving factors exist at different stages of IPF. For example, the molecular mechanisms differ significantly between stable IPF and acute exacerbation of IPF AE-IPF [48]. Therefore, future studies should further analyze the molecular characteristics of IPF in a stage-specific or stratified manner to gain a more comprehensive understanding of its dynamic pathological processes. Second, limitations at the data level also need attention. This study primarily relied on transcriptomic and genomic data but did not directly measure the expression levels of candidate proteins in the plasma or lung tissues of IPF patients. This absence may prevent validation of the consistency between transcriptional levels and protein expression, limiting the clinical translational potential of the identified biomarkers. Future research should incorporate proteomics or immunoassay-based experimental validation to improve the reliability of the findings. In the directionality test for MR, we only applied the Steiger filter method to ensure the correct direction of inference but did not use bidirectional MR to verify reverse causal relationships. Bidirectional MR could more robustly exclude the possibility of reversed causality.

## 5. Conclusions

This study integrated a set of key genes driving IPF, which are involved in multiple pathways, such as metabolism, immune response, and inflammatory processes. Through validation using machine learning models, we further confirmed the diagnostic potential of these genes. Single-cell analysis highlighted the critical roles of fibroblasts, endothelial cells, epithelial cells, macrophages, and DCs in IPF. These genes may form a molecular network facilitating communication between different cell types and contribute to the pathways driving the transition of cells from normal phenotypes to fibrotic phenotypes. By integrating multidimensional insights from genetics, immunology, and cell biology, this study provides a clear “roadmap” for targeted therapies, further advancing IPF research toward precision medicine based on molecular mechanisms.

## Data Availability

The datasets used in this study are available in online repositories, with the accession numbers and repository URLs provided in the main text.

## Abbreviations

The following abbreviations are used in this manuscript:

IPF	Idiopathic pulmonary fibrosis
ILD	Interstitial lung diseases
ECM	Extracellular matrix
GWAS	Genome-wide association studies
IVW	Inverse variance weighted
IV	Instrumental variable
MR	Mendelian randomization
FDR	False discovery rate
DEGs	Differentially expressed genes
iLISI	Local inverse Simpson’s index
